# Evaluation of the Relationship Between Dietary Inflammatory Index, MIND Diet Score, Some Serum Parameters, and Depression Nutritional Status in Adult Women Diagnosed With Multiple Sclerosis

**DOI:** 10.1002/fsn3.70722

**Published:** 2025-08-18

**Authors:** Fatma Elif Eroğlu, Gürdal Orhan, Berna Arlı, Hatice Gül Hatipoğlu, Nevin Sanlier

**Affiliations:** ^1^ Department of Nutrition and Dietetics, Gülhane Health Sciences Faculty University of Health Sciences Ankara Turkey; ^2^ Ankara Bilkent City Hospital Ankara Turkey; ^3^ Department of Nutrition and Dietetics, School of Health Sciences Ankara Medipol University Ankara Altındag Turkey

**Keywords:** depression, dietary inflammatory index, MIND diet score, multiple sclerosis quality of life, nutrition

## Abstract

This study aims to evaluate the relationship between some serum parameters, the dietary inflammatory index (DII), quality of life, depression, and nutritional status in women diagnosed with multiple sclerosis (MS). A total of 153 women aged 20–50 residing in Ankara participated in the study, including 73 MS patients and 80 in the control group. The study assessed general characteristics, anthropometric measurements, the Multiple Sclerosis Quality of Life Scale (MSQOL‐54), the Beck Depression Inventory‐21 (BDI), MIND Diet Score (MIND), a 3‐day food consumption record, the Healthy Eating Index‐2015 (HEI‐2015), DII, and biochemical findings. The average waist circumference in the MS group (84.5 ± 12.22 cm) was significantly higher than that of the control group (80.5 ± 10.60 cm) (*p* < 0.05). The average BDI score was 12.9 ± 9.66 points in the MS group and 7.9 ± 6.16 points in the control group, with the MS group scoring significantly higher (*p* < 0.05). The mean MIND diet score in the MS group (6.3 ± 1.90) was significantly lower (*p* < 0.05) compared to the control group (6.9 ± 1.71). The mean DII and HEI‐2015 scores were lower in the control group (2.2 ± 2.34 and 44.6 ± 9.86, respectively) compared to the MS group (2.4 ± 2.43 and 44.8 ± 12.69). In the MS group, 64.2% had a poor diet, whereas 72.5% of the control group had a poor diet. In the control group, 27.5% had a diet needing improvement compared to 34.6% in the MS group. This study provides detailed insights into anthropometric measurements, serum parameters, depression, and especially nutritional status in MS patients. Given the significantly low MIND diet scores in individuals with MS, implementing individualized medical nutrition therapy would be beneficial. This approach could enhance nutritional status, improve diet quality, and raise awareness about healthy and balanced nutrition for individuals with MS.

## Graphical abstract

MS group exhibited higher waist circumference (84.5 ± 12.2 cm vs. 80.5 ± 10.6 cm), higher BDI scores (12.9 ± 9.7 vs. 7.9 ± 6.2), and lower MIND adherence (6.3 ± 1.9 vs. 6.9 ± 1.7) than the Control group. Poor diet remained high in both groups (MS group 64.2%, Control group 72.5%).

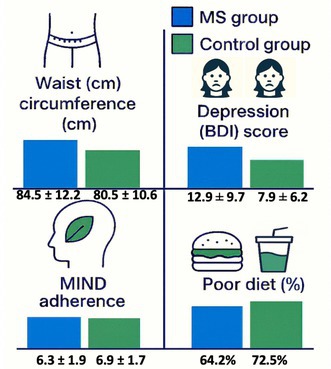

## 1 Introduction

Multiple sclerosis (MS) is a chronic autoimmune disease of the central nervous system. The disease results from demyelination, inflammation, and axonal damage in the brain (Housley et al. 2015) and generally affects young adults between the ages of 20–40, with a higher prevalence in women, two to three times more than in men (Thompson et al. 2018). It is thought that genetic and environmental factors contribute to the development of MS. Environmental factors such as smoking, Epstein–Barr virus (EBV) infection, obesity, decreased vitamin D levels, and working night shifts are known to increase the risk of MS (Olsson et al. 2017).

Eating habits and lifestyle are believed to affect the pathogenesis of MS. The effectiveness of nutritional supplements and special diets in medical nutrition therapy for MS is not well established. However, inadequate and unbalanced diets, in addition to genetic and immunological factors, can negatively affect the inflammatory process (Stoiloudis et al. 2022). Studies have shown that including vitamin D supplements, energy restriction, a semi‐vegetarian diet, or nutritional supplements (such as fish oil, lipoic acid, omega‐3 polyunsaturated fatty acids, resveratrol, and multivitamin complexes) in the diets of MS patients causes significant changes in neurological symptoms and may reduce autoimmune inflammation in MS (Fitzgerald et al. 2015). Another study suggested that increasing fiber‐rich food intake and reducing animal fat consumption positively affect MS pathophysiology (Bagur et al. 2017). High sodium levels, often found in Western‐style diets, are associated with MS. It is assumed that lower salt intake is beneficial, making Western‐style diets a risk factor for MS (Zielińska and Michońska 2023).

The Mediterranean diet, which emphasizes high consumption of olive oil, fruits, vegetables, nuts, and whole grains, moderate intake of fish and poultry, low‐fat dairy products, and minimal red meat, is associated with a reduced risk of MS. Due to its positive effects on chronic diseases, the Mediterranean diet is recommended for MS patients (Esposito et al. 2021). Additionally, nutritional models and strategies are being developed for the management of diseases like MS. For example, the Mediterranean–DASH (Dietary Approaches to Stop Hypertension) Intervention for Neurodegenerative Delay (MIND) diet combines elements of the Mediterranean and DASH diets. Studies have shown a negative association between the MIND diet score and the likelihood of having MS, especially with increased intake of green leafy vegetables and other vegetables (Noormohammadi et al. 2022).

As in other autoimmune and inflammatory diseases, antioxidants play a crucial role in reducing free radical damage and inflammation in MS patients. Thus, antioxidant‐rich anti‐inflammatory diets are recommended. The dietary inflammatory index (DII) assesses the inflammatory potential of a diet based on various nutrients, spices, and bioactive compounds related to health outcomes, including inflammatory cytokines and chronic diseases. The DII classifies 45 dietary components as either pro‐ or anti‐inflammatory. High positive DII scores indicate a more pro‐inflammatory diet, while high negative scores indicate a more anti‐inflammatory diet (Cavicchia et al. 2009).

The importance of medical nutrition therapy in the onset and treatment of MS is emphasized, though clinical intervention studies on nutrition in MS treatment are limited, resulting in a lack of strong evidence. Despite the significant role of diet in MS treatment, there is no MS‐specific nutrition model yet (Solsona et al. 2024).

Depression is prevalent among MS patients, with an estimated 35% of patients affected (Boeschoten et al. 2017). The exact cause of depression in MS is unknown. However, a significant negative relationship has been found between depression levels and cognitive performance in MS patients (Jorissen et al. 2017). Given that MS is often diagnosed in young adults, providing psychological support to those at risk is essential to understanding the relationship between depression and cognitive impairment in MS patients.

In light of this data, this study was conducted to investigate serum levels, MIND diet score, DII, quality of life, depression, anthropometric measurements, and nutritional status of women diagnosed with relapsing‐remitting multiple sclerosis (RRMS) compared to healthy women.

The hypotheses of this study are as follows:

1. Individuals with multiple sclerosis have higher BDI scores than healthy individuals.
2. Individuals with multiple sclerosis have lower MIND diet scores than healthy individuals.
3. There is a difference between the biochemical parameters of patients with multiple sclerosis compared to healthy individuals.
4. Anthropometric measurements of patients with multiple sclerosis are higher than those of healthy individuals.
5. There is a difference between the daily energy intake, other macro‐and micronutrients of patients with multiple sclerosis compared to healthy individuals.
6. There is a difference between the food groups of patients with multiple sclerosis compared to healthy individuals.

## 2 Maternal and Method

### 2.1 Participants

This study was conducted between August 2022 and January 2023, involving women aged 20–50 diagnosed with Relapsing–Remitting Multiple Sclerosis (RRMS) at the Ankara Bilkent City Hospital Neurology Clinic, as well as healthy volunteers residing in Ankara. A power analysis using the G Power 3.1.9.2 program was performed with a Type 1 error of 0.05, a Type 2 error of 0.20, and an effect size of 0.68. The analysis determined that 26 control participants and 52 experimental participants were needed, resulting in a total of 78 participants. Ultimately, 153 women (80 healthy women and 73 women with RRMS) were included in the study. All participants signed informed consent forms, indicating their voluntary participation.

Inclusion Criteria:

- Female, aged 20–50 years
- Diagnosed with RRMS
- An EDSS score of < 6.0
- Not having followed any specific diet in the last 3 months

Exclusion Criteria:

- Under 19–50 years of age
- Pregnant or having undergone surgery
- Continuous use of steroid medication except during attacks
- Following a diet before and during the study
- Having cardiac, hepatic, renal, nephropathic, pulmonary, diabetic, GI, hematological, psychiatric disease, or being male

A questionnaire developed by the researchers was administered face‐to‐face to gather personal characteristics of the participants, taking approximately 20 min to complete. Anthropometric measurements were also taken.

Ethical approval was obtained from the Ankara Medipol University Health Sciences Non‐Interventional Research Ethics Committee, with the decision dated 15/08/2022 and numbered 151.

Instruments.

Demographic and Health Information.

İndividuals;

- General information (age, education, employment status, occupation, MS disease status)
- General health and disease information (chronic diseases, family history of MS, smoking status, alcohol consumption, use of food supplements, and medications)
- Nutritional habits (number of main and snack meals consumed, whether meals are skipped, meal consumption outside the home, duration of meal consumption, daily water consumption, night‐day sleep durations)

### 2.2 Anthropometric Measurements

Participants' body weight (kg), height (cm), waist circumference (cm), and neck circumference (cm) were measured by the researcher following standard procedures (Pekcan 2008). Body composition, including body fat ratio (%), body fat mass (kg), body water ratio (%), body muscle ratio (%), lean body ratio (%), lean body mass (kg), and body mass index (BMI) (kg/m^2^), was analyzed using the SC‐330 Tanita device with the bioelectrical impedance analysis (BIA) method.

### 2.3 Evaluation of Dietary Records

To determine daily energy intake and macro‐ and micronutrient intake, 3‐day food consumption records were obtained (two weekdays and one weekend day). The Food and Nutrition Photograph Catalogue was used to accurately assess portion sizes (Rakicioğlu et al. 2012). The adequacy of daily energy and nutrient intake was evaluated according to the Dietary Reference Intake Level (DRI) (Trumbo et al. 2002). The Computer‐Assisted Nutrition Program Nutrition Information System (BeBiS) 9.0 software was used to calculate daily energy, macro‐, and micronutrient amounts.

### 2.4 MIND Diet Score (15 Items)

The MIND diet score was calculated based on 15 dietary parameters, including 10 healthy food categories (e.g., green leafy vegetables, other vegetables, berries, nuts, whole grains, poultry, olive oil, red wine) and five unhealthy categories (e.g., red meat products, butter and margarine, cheese, pastries and sweets, fast foods). Participants received scores from 0 to 15 based on their dietary habits, with higher total scores indicating better compliance with the MIND diet (Morris et al. 2015; Noormohammadi et al. 2022).

### 2.5 Healthy Eating Index‐2015 (HEI‐2015)

Participants were scored based on their food consumption records, with scores ranging from 0 to 100. A score of ≤ 50 indicated “poor diet quality,” 51–80 indicated “needs improvement,” and > 80 indicated “good diet quality” (Krebs‐Smith et al. 2018).

### 2.6 Dietary Inflammatory Index (DII)

The DII scores of 29 nutrients were calculated based on daily intake, using the National Food Composition Database (TURKOMP) when necessary. High positive DII scores indicated a pro‐inflammatory diet, while high negative scores indicated an anti‐inflammatory diet (Cavicchia et al. 2009).

### 2.7 Beck Depression Inventory 21 (21 Items)

The BDI‐21, developed by Beck et al. (Beck et al. 1961), includes 21 items measuring emotional, somatic, and cognitive symptoms commonly seen in depression. Each item is scored from 0 to 3, with total scores ranging from 0 to 63. Scores of 0–13 indicate minimal depression, 14–19 indicate mild depression, 20–28 indicate moderate depression, and 29–63 indicate severe depression.

The cut‐off score is accepted as 17 (Esposito et al. 2021). A score of 17 and above on the scale is evaluated as “there are symptoms of depression,” and a score below 17 is evaluated as “no symptoms of depression” (Jackson‐Koku 2016).

### 2.8 Biochemical Parameters

Biochemical parameters, including glucose, total cholesterol (T‐chol), triglyceride (TG), high‐density lipoprotein cholesterol (HDL‐C), low‐density lipoprotein cholesterol (LDL‐C), iron, folate, B12, and vitamin D, were routinely measured at Ankara Bilkent City Hospital with permission.

### 2.9 Evaluation of MRI Findings

MRI scans were obtained using a 3 T GE Signa Pioneer (General Electric, Milwaukee, WI, USA) using a 16‐channel neurovascular coil. No changes were observed in the MRI hardware or software during the follow‐up period. Images were obtained as T1 and T2‐weighted sequences, 3D FLAIR, and post‐contrast T1‐weighted sequences. Images were obtained and evaluated by a neuroradiologist. Patients were divided into two groups according to the lesion load on FLAIR and T2‐weighted images. The first group consisted of patients with 20 or below lesions, and the second group consisted of patients with lesion numbers over 20 and combined. Patients were evaluated as having spinal cord lesions with localization. When the contrast increased after gadolinium injection, the presence of an acute demyelinating plaque was recorded.

### 2.10 Statistical Analysis

The statistical analysis of the data obtained from the study was performed using SPSS for Windows 22.0 package program. Descriptive statistics were evaluated with continuous variables as mean ± standard deviation or median and categorical variables as frequency and percentage values. Categorical variables were evaluated with Pearson's Chi‐Square or Fisher's Exact Probability test. Kolmogorov–Smirnov test or Shapiro–Wilk test, and Independent samples t‐test, one‐way Anova, and Tukey's multiple comparison test were applied. In cases where parametric test conditions were not met, Mann–Whitney U‐test was used, and for the evaluation of three or more independent group comparisons, Kruskal–Wallis H test and Mann–Whitney U‐test with Bonferroni correction were used. In addition, Pearson correlation coefficient (rho) or Spearman correlation coefficient (rho) was also used. All statistical analyses were evaluated by accepting *p* < 0.05 as the statistical significance level.

## 3 Results

This study was conducted on a total of 153 female individuals, 73 in the MS group (47.7%) and 80 in the control group (52.3%), aged between 20 and 50. The distribution of general and clinical information about the individuals is given in Table 1. 53.6% of the individuals are between 20 and 35 years old, and 46.4% are between 35 and 50 years old. 41.1% of the individuals in the MS group and 91.2% of the individuals in the control group have Bachelor's degrees. While 64.2% of individuals in the control group were working, 36.9% of individuals in the MS group were working. 49.3% of the individuals were diagnosed with MS for 1–5 years. The average age of the individuals diagnosed with MS was 29.2 ± 7.64 years, and the average duration of MS diagnosis was 5.5 ± 5.24 years. In the classification of the number of lesions, 5.6% of the MS group was ≤ 20, nd 64.4% of the MS group was > 20 and confluent. The average daily cigarette consumption of individuals in the MS and control groups was found to be (14.1 ± 8.91 and 10.2 ± 2.02 cigarettes/day, respectively).

**TABLE 1:** General and clinical information on individuals.

	MS group		Control group		
	(*n* = 73)		(*n* = 80)		
	*n*	%	*n*	%	
Age (year)					
20–35	39	53.4	43	53.8	
36–50	34	46.6	37	46.2	
$\bar{X}$±SD Min‐Max	(34.8 ± 8.49) (21.0–50.0)		(35.7 ± 8.02) (22.0–50.0)		
Education status					
Primary education/Secondary education	24	32.9	—	—	
High school	19	26.0	7	8.8	
Bachelor's degree	30	41.1	73	91.2	
Working status					
Yes	27	36.9	75	64.2	
No	46	63.1	5	35.8	
Marital status					
Single	47	64.4	44	55.0	
Married	26	35.6	36	45.0	
Spinal cord lesion status					
Yes	64	87.7			
No	9	12.3			
Number of lesions					
<= 20	26	35.6			
> 20 ve confluence	47	64.4			
Acute lesion status					
Yes	17	23.3			
No	56	76.7			
Diagnosis time (years)					
< 1	10	13.7			
1–5	36	49.3			
> 5	27	37.0			
MS diagnosis (years)𝑿 ± SD (Min‐Max)	5.5 ± 5.24 (0.0–18.0)				
Diagnosis age (years) 𝑿 ±SD (Min‐Max)	29.2 ± 7.64 (17.0–50.0)				
Smoking					
Yes	25	34.2	11	13.8	8.912
No	48	65.8	69	86.2	0.003*

	(*n* = 25)	(*n* = 11)	
Number of cigarettes smoked in a day (𝑿 ± SD)	14.1 ± 8.91	10.2 ± 2.02	102.00
			0.233^a^

Abbreviations: MS: multiple sclerosis, χ^2^, Chi‐square test of independence.

* *p* < 0.05.

^a^ Mann–Whitney U test.

Table 2 shows the mean, standard deviation, biochemical parameters, and anthropometric measurements classification of individuals. It was observed that the mean waist circumference of the MS group (84.5 ± 12.22 cm) was significantly higher than the mean of the control group (80.5 ± 10.60 cm) (*p* < 0.05). Waist/hip (0.80 ± 0.07), waist/height (0.49 ± 0.06), and neck circumference (31.5 ± 1.95 cm) were lower than the values of the MS group (0.83 ± 0.08, 0.51 ± 0.08, 33.2 ± 2.80, respectively) (*p* < 0.05). In the classification of waist/hip ratio, 41.1% of the MS group was at risk, and 32.0% of the control group was at risk (*p* < 0.05). The neck circumference of the control group was 29.4% at risk and 70.6% at no risk, and in the MS group, it was 42.5% at risk and 57.5% at no risk. Serum fasting glucose, triglyceride, and VLDL‐C (85.8 ± 12.57, 111.4 ± 57.38, and 22.7 ± 11.08, respectively) were higher in the MS group compared to the control group (81.6 ± 8.49, 87.0 ± 42.80, and 17.7 ± 8.34, respectively) (*p* < 0.05). Serum iron, transferrin saturation, folate, and vitamin B12 values of the MS group (74.0 ± 40.58, 5.3 ± 32.61, 8.8 ± 3.57, and 331.2 ± 189.97, respectively) were lower than the control group (88.3 ± 50.24, 31.3 ± 32.82, 18.5 ± 75.55, 395.6 ± 144.91, respectively) (*p* < 0.05).

**TABLE 2:** Mean, standard deviation, biochemical parameters, and anthropometric measurements classification of individuals.

Anthropometric measurements	MS group (*n* = 73)		Control group (*n* = 80)		MWU/t
					*p*
	$\bar{X}$± SD	(Min‐Max)	$\bar{X}$± SD	(Min‐Max)	
Body weight (kg)	66.0 ± 11.14	41.1–101.10	64.9 ± 9.55	47.7–94.2	0.648 0.518^t^
Height (cm)	163.0 ± 6.71	146.0–179.0	164.1 ± 6.30	150.0–176.0	−1.103 0.273^t^
Body mass index (BMI) (kg/m^2^)	25.1 ± 4.55	18.5–37.1	24.2 ± 3.79	16.2–35.9	2717.00 0.458^£^
Waist circumference (cm)	84.5 ± 12.22	59.0–124.0	80.5 ± 10.60	61.0–102.0	2.151 0.033*, ^t^
Hip circumference (cm)	101.1 ± 8.08	83.0–122.0	100.3 ± 7.64	81.0–117.0	0.627 0.532^t^
Neck circumference (cm)	33.2 ± 2.80	28.0–42.0	31.5 ± 1.95	27.0–36.0	1890.5 *p* < 0.001*, ^£^
Waist/hip ratio	0.83 ± 0.08	0.5–1.0	0.80 ± 0.07	0.6–0.9	2167.5 0.006*, ^£^
Waist/height ratio	0.51 ± 0.08	0.35–0.75	0.49 ± 0.06	0.3–0.6	2336.0 0.033*, ^£^
Body fat percentage (%)	30.2 ± 7.04	8.2–45.6	30.5 ± 7.09	10.8–47.6	−0.253 0.801^t^
Body fat mass (kg)	21.0 ± 7.79	9.1–44.2	23.5 ± 11.73	9.6–79.0	3197.50 0.311^£^
Biochemical Parameters					
Glukoz (mg/dL)	85.8 ± 12.57	(60.0–127.0)	81.6 ± 8.49	(67.0–107.0)	2201.00
					0.009*, ^£^
Kolesterol‐K (mg/dL)	173.5 ± 38.64	(87.0–303.0)	181.7 ± 35.35	(110.0–273.0)	3308.50
					0.156
Trigliserit (mg/dL)	111.4 ± 57.38	(11.0–308.0)	87.0 ± 42.80	(11.0–286.0)	2029.50
					0.001*, ^£^
HDL‐K (mg/dL)	57.2 ± 16.41	(27.0–109.0)	57.8 ± 11.90	(30.0–86.0)	−0.230
					0.818^t^
LDL‐K (mg/dL)	95.6 ± 28.18)	(35.0–192.0)	107.8 ± 31.87	(83.5–182.0)	−2.494
					0.014^t^
VLDL‐K (mg/dL)	22.7 ± 11.08	(8.0–62.0)	17.7 ± 8.34	(6.0–59.0)	1981.50
					< 0.001*, ^£^
Demir(ug/dL)	74.0 ± 40.58	(15.0–230.0)	88.3 ± 50.24	(25.0–37.8)	3492.50
					0.036*, ^£^
Folat (ng/L)	8.8 ± 3.57	(3.0–20.0)	18.5 ± 75.55	(4.0–685.0)	3513.00
					0.029*, ^£^
B12 (ng/L)	331.2 ± 189.97	(97.0–1712.0)	395.6 ± 144.91	(172.0–876.0)	3851.00
					< 0.001*, ^£^
Vitamin D (ng/L)	53.0 ± 29.71	(18.0–151.0)	44.6 ± 24.84	(12.0–137.0)	2400.50
					0.058^£^

Anthropometric measurements classification	*n*	%	*n*	%	χ^2^/p
Waist circumference (cm)					
No risk	25	34.2	40	50	
Yes risk	22	30.1	19	28.8	3.901
High risk	26	35.7	21	26.2	0.142
Waist/hip ratio					
No risk	43	58.9	61	73.3	4.509
Yes risk	30	41.1	19	22.7	0.034*
Neck circumference (cm)					
No risk	42	57.5	66	82.5	10.288
Yes risk	31	42.5	14	17.5	0.001*

Abbreviations: MS: multiple sclerosis; χ^2^, Chi‐square test of independence.

* *p* < 0.05.

^t^ Two independent samples t‐test.

^£^ Mann–Whitney U test.

**FIGURE 1:**
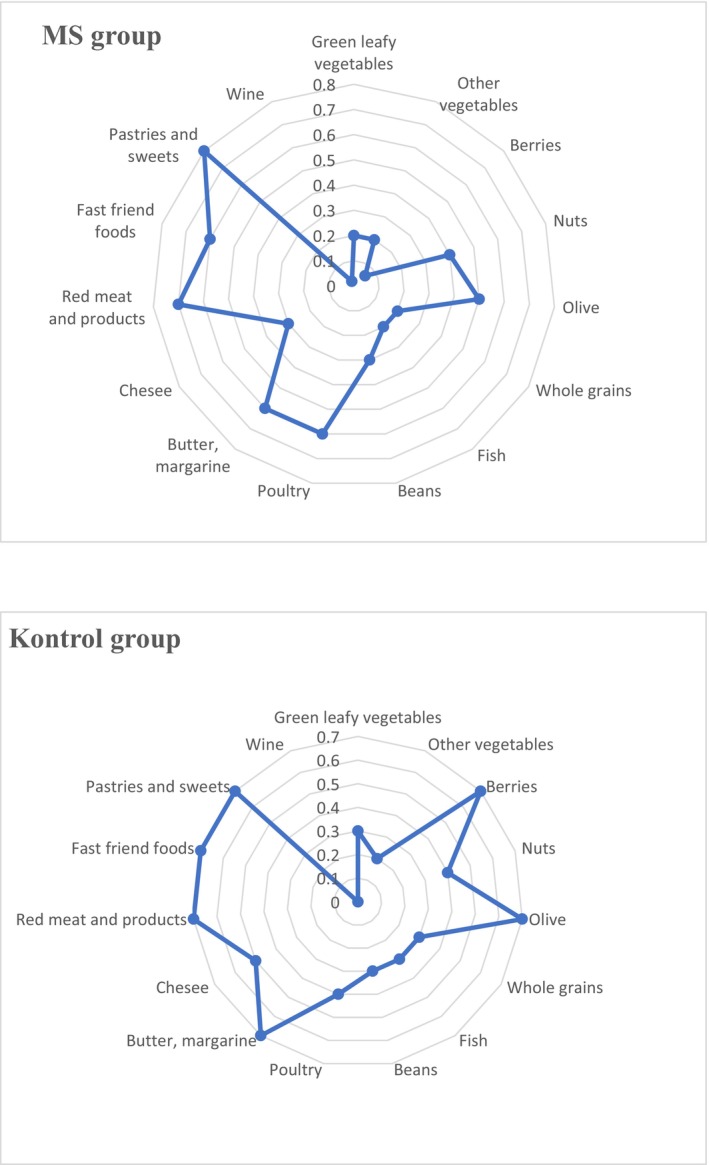
Total scores of MIND diet subcomponents of MS and Control groups. MS: Multiple Sclerosis, MIND: Mediterranean‐DASH for Neurodegenerative Delay.

The mean, standard deviation, and min‐max values of MIND, DII, HEI‐2015, and BDI scales are given in Table 3. The mean MIND diet score of the MS group was (6.3 ± 1.90) and lower than the control group (6.9 ± 1.71) (*p* < 0.05). The mean BDI score of the MS group (12.9 ± 9.65) was higher than the control group (7.98 ± 6.16) (*p* < 0.05). The total scores of the MIND diet subcomponents of the MS and control groups are given in Figure 1. The mean scores of the green leafy vegetables, whole grains, fish, butter, margarine, cheese, and fast foods components of the MS group were lower than the control group (*p* < 0.05). The mean scores of the nuts, other vegetables, beans, and red meat products components of the MS group and the control group were similar (*p* > 0.05). The food component scores of the control group for berries, olive, and cheese were higher than those of the MS group (*p* < 0.05), while the food component scores of the MS group for pastries and sweets were lower than those of the control group (*p* < 0.05).

**TABLE 3:** Mean, standard deviation, and min‐max values of MIND, DII, HEI‐2015, and BDI scales of individuals.

Scales	MS group (*n* = 73)	Control group (*n* = 80)	MWU/t
	$\bar{X}$± SD (Min‐Max)	$\bar{X}$± SD (Min‐Max)	*p*
MIND	6.3 ± 1.90	6.9 ± 1.71	−2.021
	(2.0–10.5)	3.0–12.0	0.045^t^, *
DII	2.4 ± 2.43	2.2 ± 2.34	0.517
	(−2.8–6.7)	(−2.2–6.7)	0.606^t^
HEI‐2015	44.8 ± 12.69	44.6 ± 9.86	0.075
	(24.6–76.0)	(21.8–68.2)	0.940^t^
BDI	12.9 ± 9.65	7.98 ± 6.16	2064.00
	(80.0–36.0)	(0.0–32.0)	0.002^£^, *

Abbreviations: BDI, Beck depression inventory; DII, diet inflammatory index; HEI‐2015, Healthy eating index‐2015; MIND, Mediterranean‐DASH for neurodegenerative Delay.

^t^ Two independent samples t‐test.

^£^ Mann–Whitney U test.

* *p* < 0.05.

The mean and standard deviation values of MSQOL‐54 scale component scores according to BDI classification are shown in Table 4. The mean scores of MSQOL‐54 scale components (physical health composite, physical function, role limitations‐physical, pain, energy/fatigue, social function, health perception, health distress, mental health composite, emotional well‐being, health distress, cognitive function and overall quality of life) were found to be higher in individuals with minimal depression (73.6 ± 16.55, 13.3 ± 3.30, 12.6 ± 6.47, 8.1 ± 2.50, 7.1 ± 2.26, 9.6 ± 2.15, 10.2 ± 3.35, 7.8 ± 2.46, 67.1 ± 17.48, 19.5 ± 4.09, 9.9 ± 3.13, 10.5 ± 3.64, and 12.6 ± 3.19, respectively) than in individuals with mild, moderate, and severe depression (*p* < 0.05).

**TABLE 4:** Mean and standard deviation values of MSQOL‐54 scale component scores according to BDI classification of individuals.

MSQOL‐54 scale components	BDI Classification				KW *p*/Tukey
	0–9 (Minimal depression)	10–16 (Mild depression)	17–29 (Moderate depression)	30–63 (Severe depression)	
	(*n* = 29)	(*n* = 19)	(*n* = 21)	(*n* = 4)	
	$\bar{X}$± SD (Min‐Max)	$\bar{X}$± SD (Min‐Max)	$\bar{X}$± SD (Min‐Max)	$\bar{X}$± SD (Min‐Max)	
Physical health composite	73.6 ± 16.55^a^ (26.0–97.4)	62.7 ± 7.08 (34.8–91.5)	49.5 ± 14.41^b^ (26.1–78.3)	34.7 ± 26.61^c^ (18.1–74.4)	24.474 (a‐b), (a‐c) < 0.001*
Physical function	13.3 ± 3.30^a^ (5.1–17.0)	12.6 ± 3.70 (6.8–17.0)	10.2 ± 4.02^b^ (3.4–17.0)	6.1 ± 6.29 (0.8–15.3)	11.408 **(a‐b) 0.010** *
Role limitations‐physical	12.6 ± 6.47^a^ (0.0–17.0)	9.1 ± 7.26 (0.0–17‐0)	5.8 ± 5.93^b^ (0.0–17.0)	6.1 ± 6.29 (0.8–15.3)	12.234 **(a‐b) 0.007** *
Pain	8.1 ± 2.50^a^ (1.6–11.0)	7.7 ± 2.26 (4.2–11.0)	5.8 ± 2.10^b^ (2.5–9.53)	4.2 ± 8.50 (0.8–15.3)	15.666 **(a‐b) 0.001** *
Energy/fatigue	7.1 ± 2.26^a^ (0.0–11.0)	5.4 ± 1.67 (4.8–8.16)	4.0 ± 2.04^b^ (0.9–8.6)	3.7 ± 2.08^c^ (1.9–6.9)	24.120 **(a‐b), (a‐c) < 0.001** *
Social function	9.6 ± 2.15^a^ (4.0–17.0)	9.2 ± 1.52 (7.0–12.0)	7.3 ± 2.65^b^ (3.0–12.0)	5.0 ± 2.93^c^ (2.0–9.0)	16.159 **(a‐b), (a‐c) < 0.001** *
Health perceptions	10.2 ± 3.35^ab^ (4.2–17.0)	8.4 ± 3.44 (2.5–14.4)	6.4 ± 2.55^a^ (0.8–11.9)	4.2 ± 1.20^b^ (3.4–5.9)	20.531 **(a‐b), (a‐c) < 0.001** *
Health distress	7.8 ± 2.46^a^ (2.2–11.0)	5.7 ± 2.59 (2.2–11.0)	45.1 ± 15.54^b^ (25.3–80.44)	3.0 ± 1.87^c^ (1.1–5.50)	18.053 **(a‐b), (a‐c) < 0.001** *
Sexual function	4.6 ± 3.52 (0.0–8.0)	4.1 ± 3.15 (0.0–8.0)	4.4 ± 3.00 (0.0–8.0)	3.8 ± 2.79 (2.0–8.0)	1.2080.794
Mental health composite	67.1 ± 17.48^a^ (38.2–92.5)	50.9 ± 18.99^b^ (25.3–86.3)	45.1 ± 15.54^c^ (25.3–80.4)	34.6 ± 13.69^d^ (23.5–54.6)	20.717 **(a‐b), (a‐c), (a‐d) < 0.001** *
Role limitation‐emotional	16.5 ± 9.78 (0.0–24.0)	11.3 ± 11.09 (0.0–8.0)	10.2 ± 9.17 (0.0–24.0)	6.0 ± 7.65 (0.0–16.0)	7.140 0.060
Emotional well‐being	19.5 ± 4.09^a^ (11.6–26.6)	15.7 ± 4.31^b^ (9.2–24.3)	13.4 ± 4.04^c^ (16.9–22.04)	12.7 ± 2.50^d^ (10.4–16.2)	22.821 **(a‐b), (a‐c), (a‐d) < 0.001** *
Health distress	9.9 ± 3.13 (2.8–14.0)	7.3 ± 3.30 (2.8–14.0)	6.7 ± 3.18^a^ (2.1–14.0)	3.8 ± 2.39^b^ (1.4–7.0)	18.053 **(a‐b) < 0.001** *
Cognitive function	10.5 ± 3.64^a^ (1.5–15.0)	8.9 ± 4.02 (0.7–15.0)	6.5 ± 3.33^b^ (2.2–14.4)	3.7 ± 1.93^c^ (1.5–6.0)	17.820 **(a‐b), (a‐c) < 0.001** *
Overall quality of life	12.6 ± 3.19^a^ (7.2–18.0)	0.7 ± 3.27 (6.3–16.5)	9.4 ± 2.20^b^ (6.3–14.4)	9.0 ± 1.03^c^ (8.1–9.9)	16.948 **(a‐b), (a‐c) < 0.001** *
Change in health	59.4 ± 27.88 (25.0–100.0)	51.3 ± 29.4 (0.0–100.0)	48.8 ± 27.9 (0.0–100.0)	43.7 ± 23.93 (25.0–75.0)	2.402 0.493
Satisfaction with sexual function	57.7 ± 44.87 (0.0–100.0)	59.2 ± 44.2 (0.0–100.0)	53.5 ± 31.9 (0.0–100.0)	50.0 ± 0.00 (50.0–100.0)	1.785 0.618

*Note:* a, b, c, Different characters on the same line show the difference between groups.

Abbreviations: KW, Kruskal–Wallis test; MS, multiple sclerosis; MSOQL‐54: multiple sclerosis quality of life‐54.

* *p* < 0.05.

The relationship between age, age at MS diagnosis, MSQOL‐54 Scale component scores of individuals, BMI, MIND, DII, HEI‐2015, and BDI is shown in Table 5. A negative relationship was found between age and physical health composite, role limitations‐physical, pain, energy/fatigue, social function, health perception, role limitation‐emotional, emotional well‐being, and overall quality of life, and a negative relationship was found between age at MS diagnosis and physical health composite, role limitations‐physical, pain, energy/fatigue, social function, health perception, health distress, mental health composite, role limitation‐emotional, emotional well‐being, health distress, and overall quality of life (*p* < 0.05). A negative correlation was found between BMI and composite physical health, physical health limitations due to physical problems, and pain (*p* < 0.05). In addition, positive correlations were found between MIND diet score and SYI‐2015 in the MS group (*p* < 0.05), and negative correlations were found between MIND diet score and DII and BDI in the MS group (*p* > 0.05). In the MS group, a positive but insignificant correlation was found between DII and BDI (*p* > 0.05), and a negative correlation was found between DII and HEI‐2015 (*p* < 0.05).

**TABLE 5:** Relationship between age, age at MS diagnosis, MSQOL‐54 Scale components scores of Individuals, and BMI, MIND, DII, HEI‐2015, and BDI.

MSQOL‐54 scale components	Age		Age at MS diagnosis		BMI	
	*r*	*p* ^a^	r	*p* ^a^	*r*	*p* ^a^
Physical health composite	−0.348	**0.003** *	−0.317	**0.006** *	−0.269	**0.021** *
Physical function	−0.275	**0.019** *	−0.183	0.120	−0.265	**0.023** *
Role limitations‐physical	−0.342	**0.003** *	−0.367	**0.001** *	−0.302	**0.009** *
Pain	−0.323	**0.005** ^b^, *	−0.303	**0.009** *	−0.280	**0.016** *
Energy/fatigue	−0.342	**0.003** ^b^, *	−0.338	**0.003** *	−0.212	0.072
Social function	−0.402	**< 0.001** *	−0.296	**0.011** *	−0.201	0.088
Health perceptions	−0.330	**0.004** *	−0.272	**0.020** *	−0.120	0.311
Health distress	−0.167	0.157	−0.257	**0.028** *	−0.047	0.693
Sexual function	0.032	0.788	0.134	0.260	−0.015	0.899
Mental health composite	−0.284	**0.015** *	−0.346	**0.003** *	−0.170	0.150
Role limitation‐ emotional	−0.243	**0.038** *	−0.269	**0.021** *	−0.215	0.067
Emotional well‐being	−0.232	**0.048** *	−0.277	**0.018** *	−0.192	0.103
Health distress	−0.166	0.161	−0.255	**0.029** *	−0.048	0.687
Cognitive function	−0.053	0.658	−0.0.118	0.321	−0.145	0.222
Overall quality of life	−0.312	**0.007** *	−0.277	**0.018** *	−0.109	0.357
Change in health	0.003	0.979	0.035	0.772	−0.066	0.579
Satisfaction with sexual function	0.047	0.691	0.078	0.514	0.048	0.688

	DII		HEI‐2015		BDI		MIND	
	*r*	*p* ^a^	*r*	*p* ^a^	*r*	*p* ^a^	*r*	*p* ^a^
MIND	1.000							
DII	−0.122	0.305	1.000					
HEI‐2015	0.236	**0.045** *	−0.302	0.009*	1.000			
BDI	−0.021	0.860	0.074	0.533	0.002	0.984		

Abbreviations: DII: diet inflammatory index; HEI‐2015, Healthy Eating Index‐2015; MS, multiple sclerosis; MSOQL‐54: multiple sclerosis quality of life‐54.

^a^ Spearman correlation.

^b^ Pearson correlation.

* *p* < 0.05.

Individuals' energy, macronutrient, and micronutrient intakes and food groups with different scientific organizations' recommendations are presented in Table 6. The daily energy of the MS group was determined as 1099.1 ± 380.5 kcal, while the control group was determined as 1148.7 ± 386.76 kcal (*p* > 0.05). The energy, carbohydrate (%), and fiber (g) of the control group were higher than those of the MS group, and the differences in carbohydrate (%), fat (%), and MUFAs (%) intakes were found to be statistically significant (*p* < 0.05). The daily average intake of all vitamins and minerals was found to be higher in the MS group than in the folate control group, while the daily intake of vitamin B12 and zinc was found to be lower (*p* < 0.05). The average consumption of milk and dairy products, red meat, white meat, and total meat (g/d) was higher in the control group (26.4 ± 35.07, 39.9 ± 25.09, 31.3 ± 35.40 and 122.0 ± 43.08, respectively) than in the MS group (9.63 ± 18.71, 17.2 ± 19.30, 21.3 ± 26.27, and 93.9 ± 50.64, respectively) (*p* < 0.05).

**TABLE 6:** Individuals' energy, macronutrient, and micronutrient intakes and food groups with different scientific organizations' recommendations.

		MS Group			Control Group						MS Group		Control Group		
Energy, macronutrient, micronutrient	DRI Recommendations	$\bar{X}$±SD (Min‐Max)	Median (IQR)	Meeting the requirement	$\bar{X}$±SD (Min‐Max)	Median (IQR)	Meeting the requirement	MWU/t p	Food groups (g)	Recommendation^a^	Approximate serving size	$\bar{X}$ ± SD	Approximate serving size	$\bar{X}$ ± SD	MWU/t p
Energy (kkal/day)		1099.1 ± 380.56	1030.2		1148.7 ± 386.76	1129.4		3225.0	Dairy product	3 serving/day	0.04 serving/day	9.6 ± 18.71	0.11 serving/day	26.4 ± 35.07	3922.00
	—	(452.1–2650.6)		—	(389.6–2583.0)		—	0.265^£^	Milk						**< 0.001** *
Carbohydrate (%)	% 45–65^1^	43.8 ± 7.90	45.0	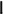	39.8 ± 8.8.43	40.00	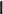	3.010			0.5 serving/day	28.4 ± 19.52	0.5 serving/day	28.9 ± 28.75	2691.50
		(21.0–59.0)			(18.0–59.0)			**0.003** ^t^, *	Cheese						0.402
Protein (%)	% 10–35 ^1^	15.2 ± 2.83	15.0	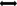	16.7 ± 2.88	16.0	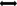	3756.00			0.3 serving/day		0.3 serving/day	62.4 ± 53.62	2659.50
		(10.0–24.0)			(12.0–26.0)			**0.002** ^£^, *	Yoghurt			74.3 ± 63.64			0.341
Dietary fat (%)	% 20–35^1^	40.5 ± 7.67	39.0	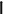	43.1 ± 7.58	43.0	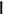	3510.00						117.8 ± 78.19	2999.50
		(24.0–60.0)			(26.0–66.0)			**0.031** ^£^, *	Total dairy products			112.3 ± 69.52			0.290
Saturated fat (%)	Hedef < %7 maximum%10^2^	19.6 ± 8.34	18.9	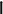	21.3 ± 8.89	19.1	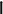	3300.00							
		(8.0–48.0)		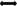	(4.2–48.4)		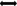	0.165^£^	Meat, Eggs, Legumes, Nuts						4539.50
Monounsaturated fatty acids (%)	%15–20^2^	16.9 ± 6.68	15.5		19.3 ± 7.55	19.4		3512.00	Red meat	¾ serving	0.9 serving/day	17.2 ± 19.30	2 serving/day	39.9 ± 25.09	**< 0.001** *
		(4.9–39.6)			(4.3–44.6)			**0.031** ^£^, *							3110.50
Polyunsaturated fatty acids (%)	%3–10^2^	9.6 ± 5.33	8.99	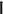	10.9 ± 4.73	10.1	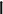	3449.50	Seafood (fish)		0.03 serving/day	4.7 ± 18.06	0.06 serving/day	6.7 ± 17.69	0.214
		(2.0–38.5)			(2.9–27.8)			0.053^£^							3420.50
Dietary fiber		11.8 ± 4.32	11.4	47.2	11.4 ± 4.64	11.1	45.6	0.645	White meat (poultry)		0.5 serving/day	21.3 ± 26.27	0.8 serving/day	31.3 ± 35.40	**0.058** *
	25 g/day^2^	(3.5–25.6)			(4.2–27.3)			0.520^t^							0.163
Thiamine(mg)	1.1	0.5 ± 0.18	0.5	45.4	0.5 ± 0.53	0.2	45.4	−0.556							2590.00
		(0.16–1.3)			(80.2–1.5)			0.579^t^	Egg	2^1/2^ serving/week	0.3 serving/day	34.3 ± 26.12	0.3 serving/day	28.3 ± 23.03	0.227
Riboflavin(mg)	1.1	0.8 ± 0.30	0.7	63.6	0.8 ± 0.37	0.8	72.7	3137.50							3234.50
		(0.2–2.0)			(0.2–2.5)			0.427^£^	Legumes	3 serving/week	0.1 serving/day	8.1 ± 19.87	0.1 serving/day	9.5 ± 18.77	0.219
Niacin(mg)	14	7.8 ± 3.18	8.0	55.7	9.7 ± 3.80	9.6	69.2	−3.288							3044.50
		(1.7–17.7)			(1.2–27.3)			**0.001** ^t^, *	Nuts	½ serving/day	0.2 serving/day	3.2 ± 6.41	0.2 serving/day	3.1 ± 4.94	0.618
Folat(mcg)	400	196.1 ± 69.47	185.4	46.2	192.7 ± 74.79	178.2	48.1	2901.00							3909.00
		(53.0–381.6)			(68.0–471.7)			0.664^£^	**Total meat group**			93.9 ± 50.64		122.0 ± 43.08	**< 0.001** *
Vitamin B_12_(mcg)	2.4	2.9 ± 3.50	2.2	120.8	3.7 ± 3.62	3.1	154.1	4067.00							
		(0.5–25.6)			(0.7–31.1)			**< 0.001** ^£^, *	**Bread ve Grains**						2364.00
Vitamin C(mg)	90	58.8 ± 33.96	54.3	61.8	54.0 ± 28.74	48.5	56.8	2720.00	Bread	3^1/2^–4 serving/day	2 serving/day	106.4 ± 69.04	1.7 serving/day	84.5 ± 56.00	**0.042** *
		(6.4–202.5)			(7.8–157.7)			0.465^£^							3299.00
Iron(mg)	18	6.1 ± 2.08	5.9	45.2	6.7 ± 2.52	6.40	49.6	3356.00	Grains		0.4 serving/day	35.69 ± 29.72	0.4 serving/day	39.1 ± 26.90	0.166
		(1.7–12.7)			(2.6–16.5)			0.111^£^							2785.00
Zin (mg)	8	5.7 ± 1.57	5.7	56.4	7.0 ± 2.23	6.8	69.3	−4.042	Refined Grains		2.8 serving/day	137.4 ± 96.11	2.5 serving/day	125.4 ± 74.26	0.523
		(1.7–11.8)			(2.4–16.1)			**< 0.001** ^t^, *							2471.00
									**Total bread group**			279.5 ± 123.62		249.1 ± 132.70	0.101
									**Vegetables—Fruits**						3257.00
									Vegetables	2^1/2^ serving/day	1.6 serving/day	240.8 ± 149.05	1.7 serving/day	259.1 ± 129.68	0.218
															2546.00
									Fruits	2 serving/day	0.3 serving/day	76.3 ± 81.27	0.2 serving/day	52.2 ± 59.53	0.172
															2983.50
									Total fruit, vegetable group			317.1 ± 170.00		311.4 ± 145.89	0.232

*Note:* 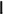: Below the recommendation, 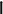: Above the recommendation, 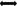: at recommendation level. ^1^DRI (Dietary reference intakes, 2001) (Nutrient Recommendations 2001). ^2^Academy of Nutrition and Dietetics (Vannice and Rasmussen, 2014).

Abbreviation: MS, multiple sclerosis.

^t^ İki bağımsız örneklem t testi.

^£^ Mann–Whitney U test.

* *p* < 0.05.

^a^ TÜBER 2022 (Recommendations for food groups are taken from the Türkiye Nutrition Guideline).

Multiple linear regression analysis for individuals DII with HEI‐2015, composite physical health, and age variables is shown in Table 7. As a result of multiple linear regression analysis of combined physical health and HEI‐2015 with DII, model significance was tested (F(2,70) = 7.364, *p* = 0.001). It was seen that as the DII value increased in the MS group, combined physical health and HEI‐2015 decreased. It was concluded that 16.4% of the change in DII score could be defined with HEI‐2015 and combined physical health. As a result of multiple linear regression analysis of HEI‐2015 and age(years), DII was tested, and the model was found to be statistically significant (F(2,77) = 8.174, *p* = 0.001). It was seen that as the DII value increased in the control group, age(years) and HEI‐2015 decreased. It was concluded that 17.9% of the change in DII score could be defined with HEI‐2015 and age(years).

**TABLE 7:** Multiple linear regression analysis for individuals DII with HEI‐2015, composite physical health, and age variables.

Scales	Regression coefficient	Standardized regression coefficient	*t*	*p*	*R* ^2^
MS Group					
DII					
Constant	7.111		5.937	< 0.001	
Physical health composite	−0.038	−0.329	−3.027	0.003	0.164
HEI‐2015	−0.051	−0.275	−2.536	0.013	
Control Group					
DII					
Constant	7.837		5.454	0.000	
Age	−0.093	−0.319	−2.969	0.004	0.179
HEI‐2015	−0.052	−0.219	−2.013	0.048	

Abbreviations: MS, multiple sclerosis; *p* < 0.05; *R* ^2^, adjusted determination coefficient T test.

## 4 Discussion

Previous studies have examined the role of diet in MS, particularly its associations with inflammation and neurodegeneration. However, our study is among the first to concurrently evaluate diet quality indices such as the HEI‐2015, MIND diet, and Dietary Inflammatory Index (DII) alongside anthropometric, biochemical, and psychological parameters in adult women diagnosed with MS. While Navarrete‐Pérez et al. investigated the effects of a MIND diet‐based intervention on oxidative stress and neurotrophic biomarkers, our observational design aimed to explore broader lifestyle, dietary, anthropometric, and biochemical factors relevant to disease progression and patient well‐being (Navarrete‐Pérez et al. 2024). Given the limited number of studies integrating such comprehensive assessments in MS populations, this research addresses an important gap in the current literature.

MS often leads to reduced functional capacity, impairing daily functioning and social participation due to symptoms such as fatigue, balance problems, and mobility limitations (Cameron and Nilsagard 2018; Novotna et al. 2016). In our study, more than half of the MS participants (63.1%) were unemployed (Table 1), likely reflecting the impact of mobility‐related challenges. Smoking was significantly more prevalent among MS patients (34.2%) compared to controls (23.5%) (*p* < 0.05), consistent with findings that link smoking to increased MS risk and worse clinical outcomes, particularly among women (Alrouji et al. 2019; Choi et al. 2017; Zhang et al. 2016).

Obesity, characterized by chronic low‐grade inflammation (Verma and Hussain 2017), is also implicated in MS pathogenesis (Meyer‐Arndt et al. 2024). In our study, MS patients had a mean BMI of 25.1 ± 4.55 kg/m^2^, categorizing them as slightly overweight (Alghwiri et al. 2018; Pilutti and Motl 2016; Zamzam et al. 2019). Although total body fat percentages were similar across groups, MS patients showed significantly higher waist circumference and fat ratios, likely due to reduced physical activity levels.

In particular, waist circumference, a marker of visceral adiposity, was significantly greater in the MS group (84.5 ± 12.22 cm) than in controls (80.5 ± 10.60 cm) (*p* < 0.05), with 35.7% of MS participants classified as high‐risk. Similarly, mean neck circumference was significantly higher among MS patients (33.2 ± 2.80 cm) compared to controls (31.5 ± 1.95 cm) (*p* < 0.05), with over half considered at‐risk according to national thresholds (Bakanlığı 2019; Kumar et al. 2014; Pei et al. 2018).

Although the waist‐to‐hip ratio (WHR) is widely recognized as a more precise predictor of cardiovascular and metabolic risk, in this study, neck circumference was chosen as a practical and easily applicable indicator of upper‐body fat distribution in clinical settings, especially considering mobility limitations in MS patients. These results suggest that central adiposity may contribute to increased inflammatory burden and disease progression in MS. Genetic studies further support a significant association between higher BMI and MS susceptibility (Valsaraj et al. 2023), and obesity has been linked to worse outcomes, including accelerated disability progression and elevated inflammatory activity (Lutfullin et al. 2022). A BMI above 27 has been consistently associated with a notably increased MS risk (Alfredsson and Olsson 2019).

Compared to healthy populations, individuals with MS exhibit a higher prevalence of psychiatric comorbidities, particularly anxiety and depression (Solaro et al. 2018). Depression, characterized by fatigue and weakness, is especially common among women with MS, who often experience difficulties in maintaining daily routines, self‐care, and social engagement (Altun and Duygu 2020). In the present study, MS participants with severe depression had the lowest emotional well‐being and role limitation‐emotional scores (Table 4), highlighting the profound impact of social isolation and psychological distress.

Consistent with previous findings (Gascoyne et al. 2019; Kahraman et al. 2021; Weiland et al. 2015), the current study showed that MS patients reported a mean daily cigarette consumption of 14.1 ± 8.91 cigarettes (Table 1). The elevated smoking rates and depressive symptoms observed reinforce the interaction between nicotine dependence, psychological burden, and diminished quality of life in this population.

Sociodemographic factors such as smoking and employment status play a critical role in shaping health outcomes in MS. In this study, the MS group exhibited a significantly higher smoking prevalence (34.2%) and a markedly lower employment rate (63.1% unemployed) compared to controls. These findings align with prior evidence that smoking not only increases MS risk but also accelerates disease progression, exacerbates brain atrophy, and worsens depressive symptoms (Alrouji et al. 2019; Choi et al. 2017). Similarly, unemployment, which was notably prevalent in our study population (63.1%), may limit social interaction, disrupt daily routines, and negatively affect nutritional habits and mental well‐being. These factors collectively contribute to heightened systemic inflammation and reduced quality of life in women with MS. Previous studies have shown that higher smoking prevalence and lower employment rates in MS patients may influence inflammatory and metabolic responses, dietary patterns, and overall health outcomes. Therefore, these sociodemographic factors were considered important contextually but were not statistically controlled for in this study, which we have acknowledged as a limitation in the manuscript.

Depression remains one of the most prevalent psychiatric comorbidities in MS, adversely affecting both physical and psychological health (Arneth 2020; Rosso and Chitnis 2020). Particularly in women, depression compromises the ability to manage daily activities, maintain self‐care, and sustain social participation. Smoking, frequently reported among MS patients, has been associated with increased depressive symptoms. Notably, the BENEFIT‐11 study found that neurofilament light chain (NfL) levels, a biomarker of neuronal damage, were approximately 20% higher in MS patients who smoked, suggesting a potential link between smoking behavior and neurodegeneration (Yang et al. 2020; Cortese et al. 2020).

In the present study, MS participants with more severe depressive symptoms exhibited significantly lower scores across key quality of life domains, including social functioning, energy, and physical health (*p* < 0.001; Table 4). These results are consistent with prior studies reporting elevated fatigue, depression, and anxiety levels among individuals with MS (Fidao et al. 2021; Scherder et al. 2018), as well as the association between psychological symptoms and heightened pain perception. In the study conducted by Jellinger (2024), it was emphasized that impairments in visual memory and processing speed in individuals with MS frequently co‐occur with depression, anxiety, fatigue, and sleep disorders. Collectively, these findings underscore the critical importance of integrating psychological assessments into MS management strategies to improve health‐related quality of life outcomes.

The interplay between psychological and cognitive dysfunctions in MS may be linked to underlying biological mechanisms, particularly inflammation. Given the established role of inflammation in both MS pathogenesis and mood disorders, the inflammatory potential of the diet emerges as a crucial factor. In this study, DII scores ranged from −8.87 to 7.98, confirming the validity of the calculations (Hébert et al. 2019; Shivappa et al. 2014). Although the mean DII score was slightly higher in the MS group (2.4 ± 2.43) compared to controls (2.2 ± 2.34), this difference was not statistically significant (*p* > 0.05; Table 3). Nonetheless, the trend suggests that MS patients may follow relatively more pro‐inflammatory diets. This aligns with previous literature highlighting that specific nutrients can modulate inflammatory pathways and impact disease outcomes (Barrea et al. 2018; Cowan et al. 2020; Kotemori et al. 2020).

Large‐scale cohort studies have consistently reported an inverse association between adherence to the Mediterranean diet and DII scores (Hodge et al. 2018; Ruiz‐Canela et al. 2015). Similarly, in the present study, a negative, though nonsignificant, correlation was observed between DII and MIND diet scores, suggesting that healthier dietary patterns may lower dietary inflammation (*p* > 0.05; Table 6). Recent randomized controlled trials have also suggested a potential neuroprotective effect of Mediterranean‐style diets in MS populations (Bruce et al. 2023). Diets rich in sodium, animal fat, and fried red meat, hallmarks of the Western diet, have been associated with an elevated risk of MS, likely through pro‐inflammatory mechanisms (Riccio and Rossano 2015).

The combined use of DII and MIND diet assessments may offer a practical framework for personalized nutritional interventions. Higher DII scores indicate the need to mitigate systemic inflammation, whereas higher MIND diet scores reflect neuroprotective eating habits, including increased intake of leafy greens, berries, and fish. Understanding the dietary contributors to inflammation is crucial when interpreting DII scores and developing targeted nutritional plans. Particularly, frequent consumption of processed meats, refined sugars, and trans fats is known to significantly elevate DII scores, thereby enhancing systemic inflammation. Employing both indices could enhance dietary counseling strategies aimed at improving disease outcomes in MS patients. Translating these dietary findings into clinical practice through structured nutritional interventions may enhance overall MS management, beyond pharmacological therapies alone. Although Western diets characterized by low fiber and high processed food intake have been linked to increased MS lesion formation (Tryfonos et al. 2024), findings remain inconsistent, as a Brazilian study reported no significant impact of dietary intake on MS prognosis (da Costa Silva et al. 2019). Interestingly, in this study, daily red meat consumption was significantly lower in the MS group compared to controls (*p* < 0.05; Table 6), which might reflect greater dietary awareness postdiagnosis. Nevertheless, focusing solely on individual food items may not capture the cumulative inflammatory potential of the overall diet, emphasizing the importance of holistic dietary assessments in MS research.

Fish, a core element of the Mediterranean diet, is rich in omega‐3 fatty acids and recognized for its anti‐inflammatory effects, offering potential protective benefits in MS (Bagur et al. 2017). Incorporating foods like fish, leafy greens, and nuts may reduce the overall dietary inflammatory load and modulate disease progression. Although our study observed a positive correlation between DII and BDI scores among MS participants, this relationship did not reach statistical significance (*p* > 0.05; Table 6). Nonetheless, the trend suggests that higher dietary inflammation may contribute to depressive symptoms, potentially exacerbated by insufficient intake of anti‐inflammatory foods such as green tea, turmeric, onion, garlic, and pepper.

Changes in lipid metabolism have also been proposed as markers of chronic inflammation. Consistent with prior studies (Siddiqui et al. 2023; Zoubi et al. 2023; Jorissen et al. 2017; Marck et al. 2016; Boshra et al. 2022), individuals with MS in our study exhibited higher serum triglycerides and VLDL‐C levels compared to controls (*p* < 0.05; Table 2). These findings may reflect dietary patterns high in saturated fats, refined carbohydrates, and processed grains, contributing to a pro‐inflammatory state.

Furthermore, fasting blood glucose levels were significantly elevated in the MS group (*p* = 0.009; Table 2), aligning with evidence linking impaired glucose regulation to inflammatory cytokines such as TNF‐α and IL‐6 (Nirooei et al. 2022; Wens et al. 2015). Inadequate intake of fiber and complex carbohydrates may exacerbate this metabolic disturbance.

Regarding micronutrient status, serum iron levels were significantly lower among MS participants (*p* = 0.036; Table 2). This finding may be related to lower consumption of bioavailable iron sources like red meat and greater reliance on plant‐based proteins with reduced iron bioavailability, consistent with previous reports (Armon‐Omer et al. 2019; Matar et al. 2020).

Folate and vitamin B12 are essential cofactors for one‐carbon metabolism and myelin regeneration (Calderón‐Ospina and Nava‐Mesa 2020). Disruptions in their levels have been implicated in MS pathogenesis (Khosravi‐Largani et al. 2018). In this study, serum vitamin B12 and folate levels were significantly lower in the MS group compared to controls (*p* < 0.001 and *p* = 0.029, respectively; Table 2), potentially predisposing patients to elevated homocysteine levels, which are linked to neurological dysfunction and depressive symptoms (Triantafyllou et al. 2008). Although homocysteine was not directly assessed, previous studies have demonstrated that folic acid and vitamin B12 supplementation can improve neurological and psychological outcomes in MS (Nozari et al. 2019; Shemirani et al. 2023; Titcomb et al. 2021). The low serum B12 and folate levels observed may stem from inadequate intake of animal‐based foods, particularly red meat, as well as gastrointestinal malabsorption and food‐drug interactions, all of which are common challenges in MS management.

Vitamin D, known for its immunomodulatory role in MS, also showed suboptimal levels among participants, despite slightly higher levels in the MS group compared to controls (*p* > 0.05; Table 2). This insufficiency may reflect reduced sun exposure due to heat sensitivity, limited outdoor activity, and variability in supplementation practices (Dörr et al. 2020; Smolders et al. 2019; Butzkueven et al. 2023). These findings highlight the need for individualized supplementation strategies alongside pharmacological treatments (Schwarz and Leweling 2005; Stoiloudis et al. 2022).

Although total daily energy intake did not differ significantly between groups (*p* > 0.05; Table 6), MS participants exhibited higher body fat percentages, possibly reflecting impaired energy metabolism and oxidative stress. Consistent with previous reports (Atabilen et al. 2024), saturated fat intake exceeded DRI recommendations in both groups, with slightly lower levels observed in MS patients. However, intakes of monounsaturated and polyunsaturated fats were lower in the MS group, likely due to reduced consumption of omega‐3‐rich foods like nuts and oily fish.

Carbohydrate intake, particularly from simple sources such as white bread, rice, and pasta, was significantly higher in the MS group (*p* < 0.05; Table 6), reflecting potentially less healthy dietary patterns. Moreover, animal protein intake was significantly lower among MS participants, leading to decreased intake of key micronutrients such as vitamin B12, iron, and zinc (Bakanlığı 2022). These patterns underline the importance of promoting balanced, nutrient‐dense dietary habits among individuals with MS.

## 5 Conclusion and Recommendations

The nutritional status of individuals with MS has been shown to be associated with dietary quality, biochemical parameters, MRI findings, anthropometric measurements, and quality of life. Diet is a modifiable factor influencing systemic inflammation, which may contribute to MS development and disease progression. The DII is a validated tool for assessing the inflammatory potential of an individual's diet and can guide personalized nutritional interventions. Higher DII scores reflect greater consumption of pro‐inflammatory foods, such as red meat, saturated fats, and added sugars, while lower scores are associated with anti‐inflammatory components, including fiber, polyphenols, omega‐3 fatty acids, and antioxidants.

In the present study, MS participants demonstrated lower adherence to the MIND diet and exhibited a higher inflammatory potential in their dietary patterns. These findings highlight the need for nutritional strategies aimed at reducing dietary inflammation. We recommend that individuals with MS reduce the intake of foods associated with higher DII scores and increase the consumption of anti‐inflammatory foods, such as whole grains, vegetables, fruits, fatty fish, and polyunsaturated fatty acids. Education on healthy eating habits and personalized medical nutrition therapy may support disease management and improve quality of life in this population.

Given the frequent occurrence of depression in MS, the inclusion of tryptophan‐rich foods such as eggs, fish, and oats may help support serotonin synthesis and mood regulation. Anthropometric findings, such as increased waist and neck circumference, suggest an elevated risk of obesity, which may further contribute to systemic inflammation. The Mediterranean diet, known for its anti‐inflammatory and antioxidant properties, may offer additional benefits by preventing obesity, reducing cardiovascular risk, and supporting neurological function. Given the multifactorial nature of MS, a comprehensive nutritional assessment that considers biochemical, psychological, and anthropometric parameters is recommended before diet planning. These findings may inform the development of nutritional counseling strategies tailored to the specific needs of MS patients.

## 6 Limitation

Several limitations of this study should be acknowledged. First, the sample consisted exclusively of women recruited from a hospital setting, limiting the generalizability of the findings. Second, the cross‐sectional design precludes causal inference. As this was an observational study without a nutritional intervention, causal relationships between diet quality and biochemical or psychological outcomes could not be established. Third, potential confounding variables, including physical activity level, disease duration, medication use, and sleep quality, were not assessed or statistically controlled. These factors are known to influence metabolic and psychological outcomes and may have affected the observed relationships. Due to the constraints of the study design and data collection, adjustments for these variables could not be performed. Furthermore, although glucose, lipid profile, and vitamin levels were evaluated, more direct inflammatory biomarkers such as C‐reactive protein (CRP) and interleukins were not assessed, which should be considered a limitation. Future research should incorporate these parameters to allow for more precise and causally interpretable findings.

Additionally, variables such as gastrointestinal health and food‐drug interactions, which are particularly relevant in MS due to common medication regimens, were not evaluated. Medication side effects may influence gut microbiota, which in turn could impact both inflammatory status and overall nutritional health. Moreover, the absence of detailed MRI imaging prevented a more comprehensive evaluation of the relationship between lesion burden, diet quality, and anthropometric measurements. Longitudinal and intervention‐based studies involving larger and more diverse populations are warranted to validate and extend these findings.

To enhance scientific rigor, future studies should employ longitudinal and interventional designs, incorporate diverse populations, and utilize comprehensive clinical, biochemical, and neuroimaging assessments.

## 7 Future Perspective

When evaluating the nutritional status of individuals with MS, it is critical to adopt a comprehensive, multidimensional approach. This should integrate anthropometric measurements, detailed assessments of dietary energy and nutrient intake (both macro‐ and micronutrients), quality of life evaluations, depression status assessments, 3‐day food records, and validated dietary tools such as the Healthy Eating Index and the Dietary Inflammatory Index. Whenever feasible, the incorporation of biochemical parameters, clinical symptom assessments, and neuroimaging data can further enrich the comprehensiveness of nutritional evaluations.

Improving the nutritional status of individuals with MS holds significant potential to enhance both quality of life and disease outcomes. Accordingly, future research must urgently focus on the development of well‐designed, large‐scale prospective cohort studies and randomized controlled trials to better establish causal relationships. Investigations targeting inflammatory signaling pathways, gut microbiota modulation, oxidative stress responses, and food‐drug interactions are particularly warranted to unravel the complex interplay between nutrition, immune function, and MS pathophysiology.

In addition, mechanistic research, including in vitro, in vivo, animal models, and human clinical trials, is essential to enhance the translational relevance of nutritional interventions in MS. These studies should also consider the potential risks associated with overnutrition and excessive nutrient intake, especially in individuals with impaired metabolic homeostasis.

Ultimately, interdisciplinary and longitudinal research efforts combining nutrition science, neurology, immunology, and psychology will be pivotal in developing precision nutrition strategies aimed at the prevention, management, and progression control of MS. Future research should prioritize the development of precision nutrition strategies, tailored to individual metabolic profiles, inflammatory status, and genetic predispositions. By integrating personalized dietary interventions with clinical management, it may be possible to more effectively modulate disease progression and improve overall quality of life in individuals with MS.

## Author Contributions

**Fatma Elif Eroğlu:** conceptualization (equal), data curation (equal), investigation (equal), methodology (equal), writing – original draft (equal). **Gürdal Orhan:** data curation (equal), investigation (equal). **Berna Arlı:** data curation (equal), investigation (equal). **Hatice Gül Hatipoğlu:** data curation (equal), investigation (equal). **Nevin Sanlier:** investigation (equal), methodology (equal), supervision (equal), visualization (equal), writing – review and editing (equal).

## Ethics Statement

Ethical approval was obtained from the Ankara Medipol University Health Sciences Non‐Interventional Research Ethics Committee, with the decision dated 15/08/2022 and numbered 151.

## Conflicts of Interest

The authors declare no conflicts of interest.

## Data Availability

The datasets are available from the corresponding author upon request.
